# Supplementary material for: Blood-based DNA methylation and exposure risk scores predict PTSD with high accuracy in military and civilian cohorts
Source: BMC Med Genomics. 2024 Sep 27;17:235. doi: 10.1186/s12920-024-02002-6 (PMC11429352; doi:10.1186/s12920-024-02002-6)
Supplement: Supplementary file 1 — Supplementary Material 1. [file 12920_2024_2002_MOESM1_ESM.docx]

***Discovery Cohorts***

*Detroit Neighborhood Health*Study:

Detroit Neighborhood Health Study (DNHS) is a prospective population-based longitudinal cohort of individuals living in Detroit, Michigan [1, 2]. The Institutional Review Board of the University of Michigan and the University of North Carolina-Chapel Hill reviewed and approved this study. In DNHS, the main goal was to investigate whether genetic variations, exposure to stressful and traumatic life events, and environmental factors such as income distribution and residential segregation influence the risk of PTSD. The cohort predominantly self-identified as African American (AA); all participants were 18 or older. Informed consent was obtained at the beginning of a structured telephone interview each year between 2008 and 2013 to collect information on participants' self-reported demographics (age, gender, race), trauma exposures, PTSD symptoms, symptom severity, and childhood maltreatment. Informed consent was again obtained at specimen collection.

Exposure to lifetime traumatic event types was assessed using a survey of 19 item traumatic events, as described in previous work [3]. The cumulative traumatic burden was estimated by summing the lifetime traumatic event types [2]. PTSD was assessed according to the Diagnostic and Statistical Manual of Mental Disorders (DSM-IV) criteria [4] using the PTSD Checklist Civilian Version (PCL-C) as described in [2]. Childhood maltreatment measures were drawn from the conflict tactics scale (CTS) [5] and the Childhood Trauma Questionnaire (CTQ) [6], previously described in detail [7].

Grady Trauma Project:

The Grady Trauma Project (GTP) was to increase the understanding of the environmental risk factors concerning PTSD and major depressive disorder (MDD) in an urban low-income population of predominately African Americans [8]. The study was approved by the Institutional Review Board of Emory University School of Medicine and the Grady Health Systems Research Oversight Committee. All participants provided written informed consent, and interviews were conducted to collect data about self-reported demographics (age, gender, race), trauma exposures, and PTSD symptoms, including biospecimens.

Exposure to lifetime traumatic events was assessed using the Traumatic Events Inventory (TEI) [8, 9], and the Childhood Trauma Questionnaire (CTQ) [10] was used to evaluate childhood maltreatment such as sexual, physical, and emotional abuse. PTSD diagnosis was assessed using the Clinician‐Administered PTSD Scale (CAPS) [11, 12].

*Army Study to Assess Risk and Resilience in Servicemembers:*

The Army Study to Assess Risk and Resilience in Servicemembers (Army STARRS) is a prospective study to understand better the risk and resilience factors for suicidal ideation and its psychopathological correlates to reduce Army suicides [13]. The study was approved by the Human Subjects Committees of the Uniformed Services University of the Health Sciences for the Henry M. Jackson Foundation (the primary grantee), the Institute for Social Research at the University of Michigan (the organization collecting the data), and all other collaborating organizations.

All the study participants provided written informed consent, and self-reported data were collected on demographics (age, gender, race). Trauma burden was drawn from self-administered questions on the civilian and military-related traumatic events except Army STARRS which reported whether exposed to trauma or not in binary format. Childhood maltreatment was assessed using 15 items scale from the Army STARRS New Soldier Survey (NSS) as described in previous work [14]. PTSD diagnosis was based on PTSD Checklist (PCL) and Composite International Diagnostic Interview Screening Scales (CIDI-SC) data as described previously [15].

­­*Marine Resilience Study I&II:*

The Marine Resiliency Study (MRS) is a prospective longitudinal study of Marines deployed to Iraq or Afghanistan, focusing on factors predicting PTSD [16, 17]. The institutional review boards of the University of California San Diego, VA San Diego Research Service, and Naval Health Research Center approved the study. After informed consent, information was collected about demographics, trauma burden, PTSD, and childhood maltreatment.

The methodology is described in the previous work [17]. Briefly, age, sex, and race were self-reported demographics. Trauma burden was assessed using Life Events Checklist. PTSD was assessed before deployment and 3 and/or 6 months post-deployment using the Clinician-Administered PTSD Scale (CAPS) and the PTSD Checklist (PCL) for DSM-IV. Childhood Trauma Questionnaire (CTQ) was used to assess childhood maltreatment.

*Prospective Research in Stress-related Military Operations:*

The Prospective Research in Stress-related Military Operations (PRISMO) is a study of Dutch military personnel deployed to Afghanistan [18, 19]. The Institutional Review Board of the University Medical Center Utrecht (Utrecht, the Netherlands) approved the study.

All the study participants provided written informed consent. Trauma exposure was assessed using a deployment experiences checklist, as reported in previous work [20]. Early life traumatic exposure was assessed before age 18 using the Early Trauma Inventory-Self-Report (ETI-SR) [21]. Diagnosis for PTSD over the past four weeks (current PTSD) was using Self-Report Inventory for PTSD (SRIP) [22].

***External Cohorts:***

*Biomarkers, social, and affective predictors of suicidal thoughts and behaviors in adolescents (BEAR).* The BEAR study, described in detail elsewhere [23], involved a sample of 194 adolescents (ages 13-18) who had been hospitalized for suicidal thoughts/behaviors. Blood collection and clinical interview procedures were completed during hospitalization. Trauma exposure and symptoms of posttraumatic stress disorder (PTSD) were assessed via clinical interview with gold standard measures, specifically the Life Events Checklist for DSM-5 and the Clinician Administered PTSD Scale for Children and Adolescents for DSM-5. If participants endorsed multiple traumatic experiences, participants were asked to focus on current symptoms related to the most upsetting event. All participants were consented to the present research and procedures were approved by the Lifespan Hospitals IRB.

NCPTSD-TRACTS: This cohort consisted of participants from three individual studies: the VA Boston National Center for PTSD (NCPTSD) study [24], the PTSD & Accelerated Aging study (Wolf et al., under review), and the Translational Research Center for TBI and Stress Disorders (TRACTS) study [25]. The NCPTSD study included trauma-exposed veterans and a subset of their trauma-exposed intimate partners. The PTSD & Accelerated Aging study included Veterans who screened positive for PTSD and who were subsequently evaluated comprehensively. The TRACTS cohort consisted of post-9/11 Veterans. For all three studies, PTSD diagnostic status was determined based on the Clinician-Administered PTSD Scale (CAPS) for DSM-IV [26] or DSM5 [27] and childhood and adulthood trauma were evaluated with the Traumatic Life Events Questionnaire [28]. For this study, lifetime but not current PTSD cases and samples with 0 self-reported trauma counts (remitted cases) were excluded. DNA methylation was measured using the Illumina EPIC 850K BeadChip. The Psychiatric Genomic Consortium-PTSD Workgroup quality control pipeline was used to process the methylation data prior to the analysis (https://github.com/PGC-PTSD-EWAS/EPIC_QC). This study was approved by the VA Boston Healthcare System and Michael E. DeBakey VA Medical Center Institutional Review Boards.

PROlonGed ExpoSure and Sertraline Trial (PROGrESS): The participants included in this study were part of a larger study called the PROlonGed ExpoSure and Sertraline Trial (PROGrESS), a randomized-controlled trial carried out across four sites: VA Ann Arbor Healthcare System (VAAAHS), Ralph H. Johnson VA Medical Center (CHSVAMC), Massachusetts General Hospital (MGH), and VA San Diego Healthcare System (VASDHCS). The study involved 223 participants and aimed to assess the effectiveness of Prolonged Exposure plus placebo (PE/PLB), Sertraline plus Enhanced Medication Management (SERT/EMM), or combined treatment (PE/SERT) on Post-Traumatic Stress Disorder (PTSD). The study also examined the neurobiological predictors and potential biomarkers of treatment response, including hypothalamic-pituitary-adrenal axis (HPA), brain, and genetic/genomic biomarkers. The PROGrESS study was approved by the institutional review boards at VHAAAHS, the University of Michigan, VASDHCS, CHSVAMC, MGH, and the Department of Defense Human Research Protection Office (HRPO). Participants and clinicians were blinded to pill condition through week 24, and independent evaluators were blinded to treatment assignments for the duration of the study. The PROGrESS study methods were published in detail [29, 30]. Briefly, the study included service members or veterans of the Iraq or Afghanistan wars who had combat-related PTSD and experienced significant impairment lasting at least three months, with a Clinicians-Administered PTSD Scale for DSM-IV (CAPS-IV) score of 50 or higher. Exclusion criteria included factors related to safety and appropriateness of psychotherapy and sertraline treatment[29, 30]. In this study, individuals diagnosed with PTSD and a control group were chosen from the pre-treatment visit.

Drakenstein Child Health Study (DCHS): The Drakenstein Child Health Study (DCHS)[31], is a population-based birth cohort, with participants initially recruited in a peri-urban area of South Africa between March 2012 and March 2015. Pregnant women who were 18 years or older, between 20-28 weeks gestation, and residing in the area were enrolled from 2 public health clinics. Study visits were scheduled in conjunction with health care and immunization visits. During the first two years of the study, additional study visits occurred at 6, 12, and 24 months, and mother-infant pairs could participate in intensive biweekly follow-up during the first year. The study followed mother-child pairs from birth through childhood, collecting longitudinal measurements of risk factors, including environmental, nutritional, immunological, genetic, infectious, and maternal factors. Maternal smoking or passive smoke exposure was self-reported. A composite measure of socioeconomic status (SES), which encompassed current employment, education, household income, and an asset index, was based on previous work in the South African Stress and Health Study. Maternal mental health measures included assessments of PTSD, depression, psychological distress, and intimate partner violence (IPV) both antenatally and postnatally. For the purpose of this study, PTSD was assessed using The Mini International Neuropsychiatric Interview (MINI) [32, 33]. The DCHS was approved by the Human Research Ethics Committee (HREC) of the Faculty of Health Sciences, University of Cape Town (UCT) and by the Western Cape Provincial Research Committee.


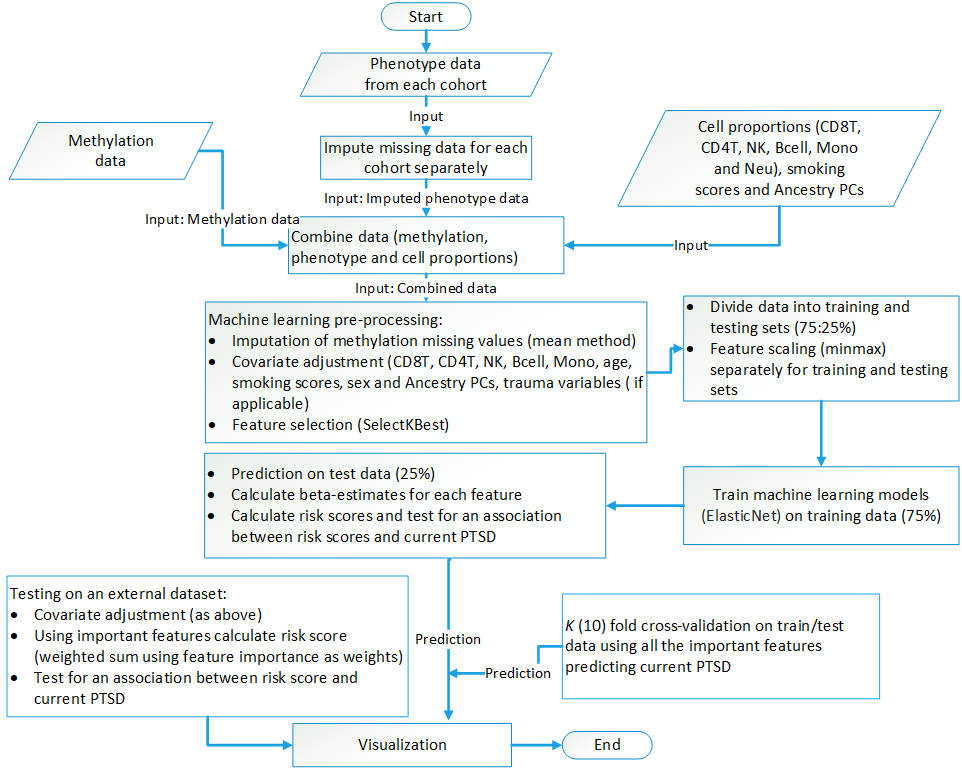


**Figure S1**: Workflow—To prepare the data for analysis, we combined methylation and phenotype data from each cohort. Data imputation and covariate adjustment was conducted separately for each cohort**,** followed by feature selection and scaling after combining data from all five cohorts. For model 3 (MoRSAE), childhood trauma and cumulative trauma variables were used in covariate adjustment, in addition to the variables described in the flow chart. We divided the data into a training and testing set, using 75% for training models and 25% for testing. We performed cross-validation using both sets. We created risk scores in the discovery cohort test set and external cohorts using weights (effect sizes) from the elastic net model Finally, we visualized the results for interpretation.


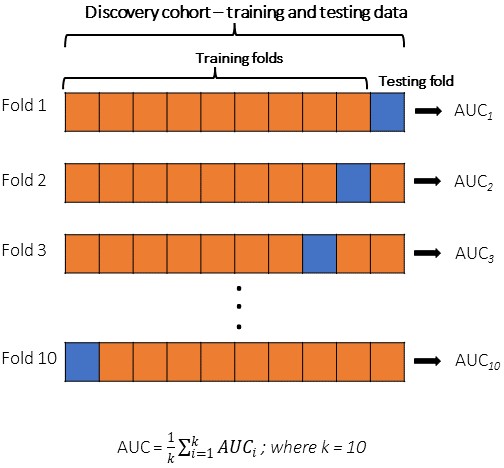


Figure S1.1: Visual representation of the 10-fold cross validation on discovery cohort.


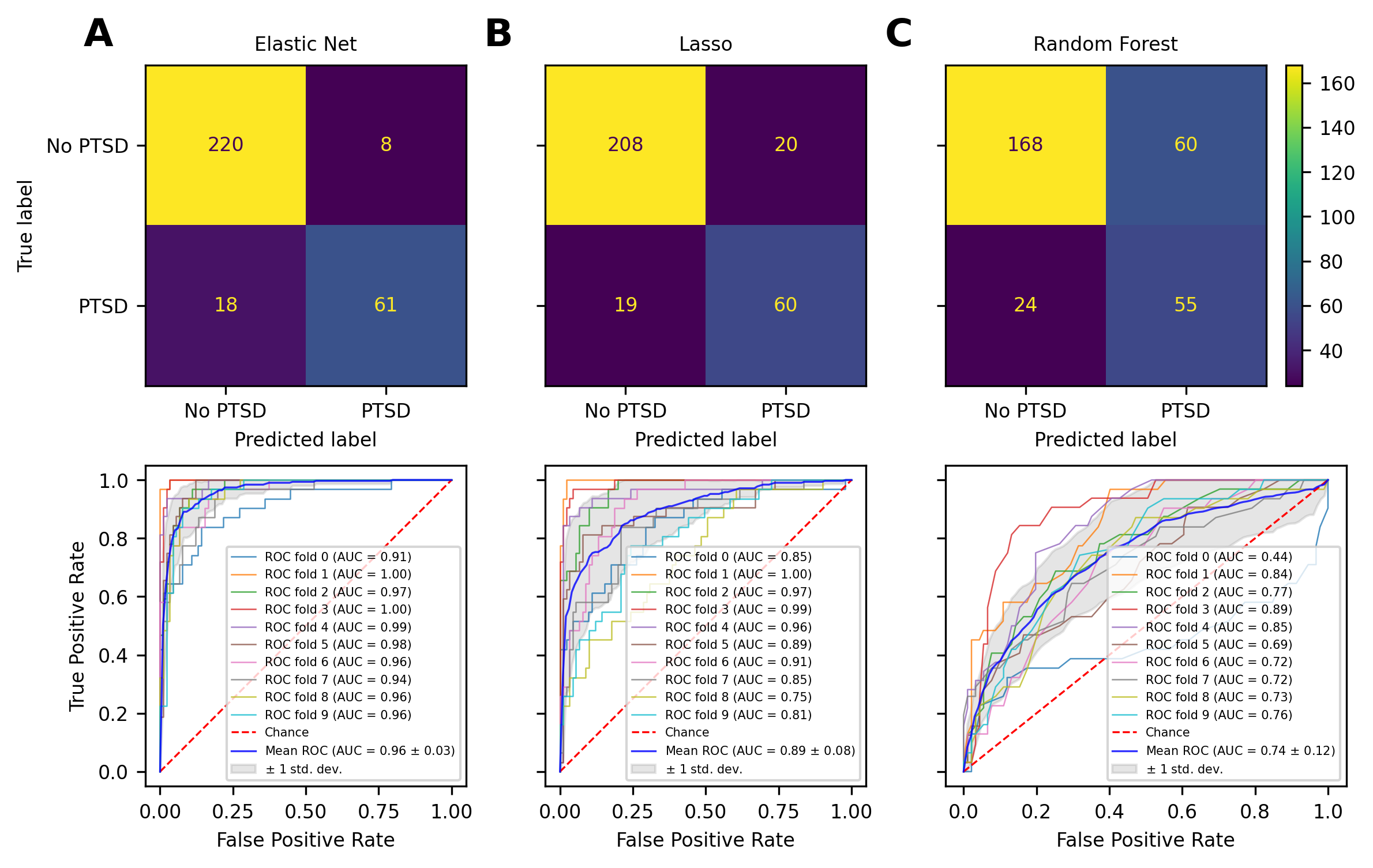


**Figure S2**: Accuracy and AUC results for three different models: Elastic Net, Lasso, and Random Forest. The Elastic Net model achieved a 92% accuracy and 96% AUC (as shown in A), while the Lasso model achieved an accuracy of 87% and an AUC of 89% (as shown in B). Finally, the Random Forest model achieved an accuracy of 73% and an AUC of 74% (as shown in C). Based on these results, it can be concluded that the Elastic Net model performed better than the Lasso and Random Forest models.


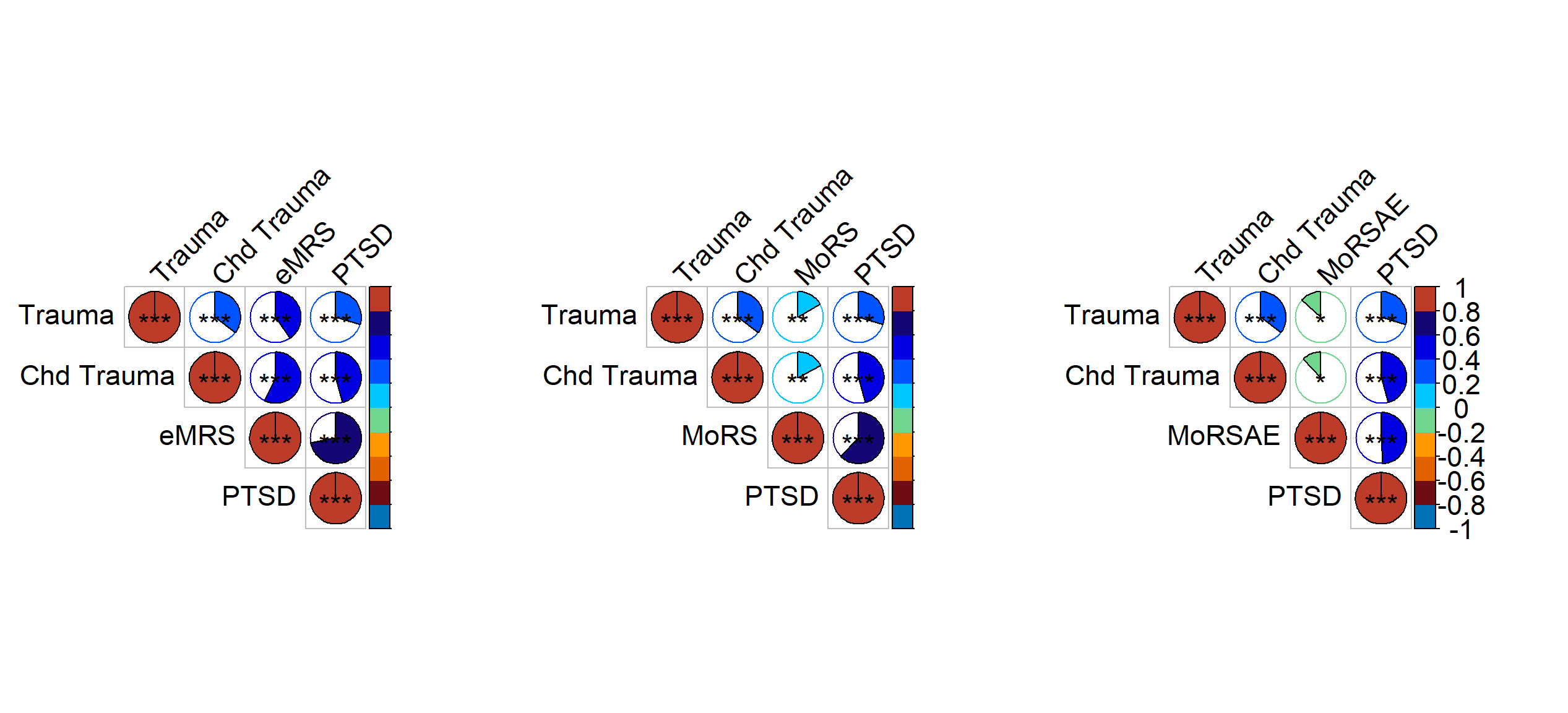
**Figure S3**: Direction of effect and strength of association among study variables in the discovery cohort test set in Models 1-3. Color and degree of fill within each circle indicate the direction of effect and strength of association between study variables, with the Asterisks indicate level of significance (*p<0.05; **p<0.01; ***p<0.001). Correlation between exposure variables and methylation risk score (eMRS) from Model 1 (left plot), methylation-only risk score (MoRS) from Model 2 (middle plot) and methylation only risk scores with adjusted exposure variables (MoRSAE) from model 3 (right plot) was assessed using Pearson's correlation. Point-Biserial correlation was used to assess correlation between continuous variables such as Trauma, Chd trauma and risk scores and binary variable (PTSD). Results show that most variables in all 3 models show a significant, positive correlation.


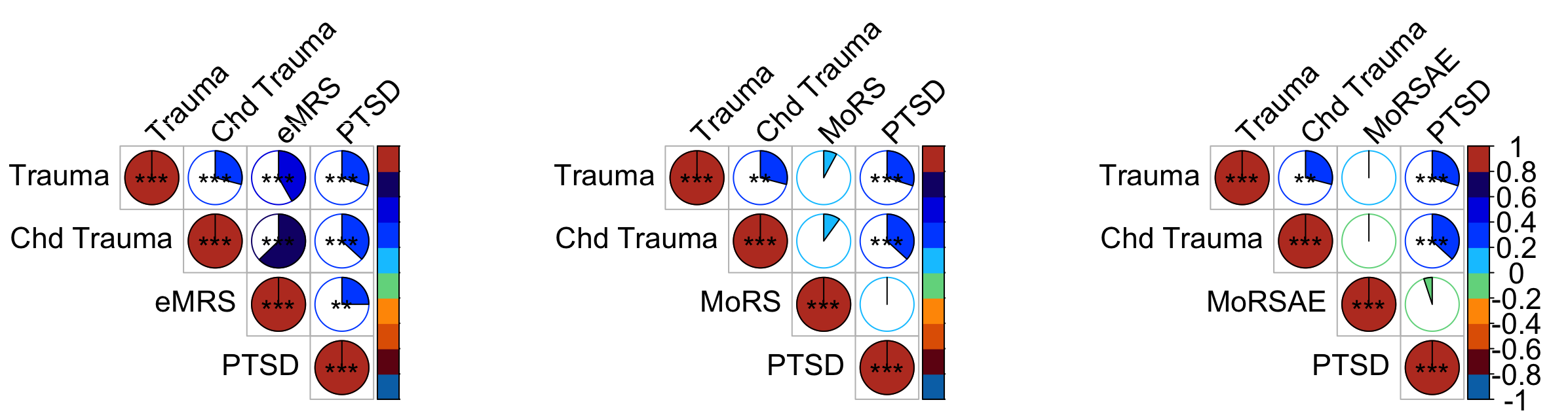
**Figure S4:** Direction of effect and strength of association among study variables in the BEAR cohort set in Models 1-3. Color and degree of fill within each circle indicate the direction of effect and strength of association between study variables, with the Asterisks indicate level of significance (*p<0.05; **p<0.01; ***p<0.001). Correlation between continuous variables was analyzed using Pearson's correlation. Point-Biserial correlation was used correlation between continuous variables and PTSD. There was a significant (p < 0.01) and positive correlation between eMRS and PTSD. Also trauma and childhood trauma (Chd Trauma) show positive and significant correlation with eMRS. MoRS and MoRSAE showed no significant correlation with PTSD.


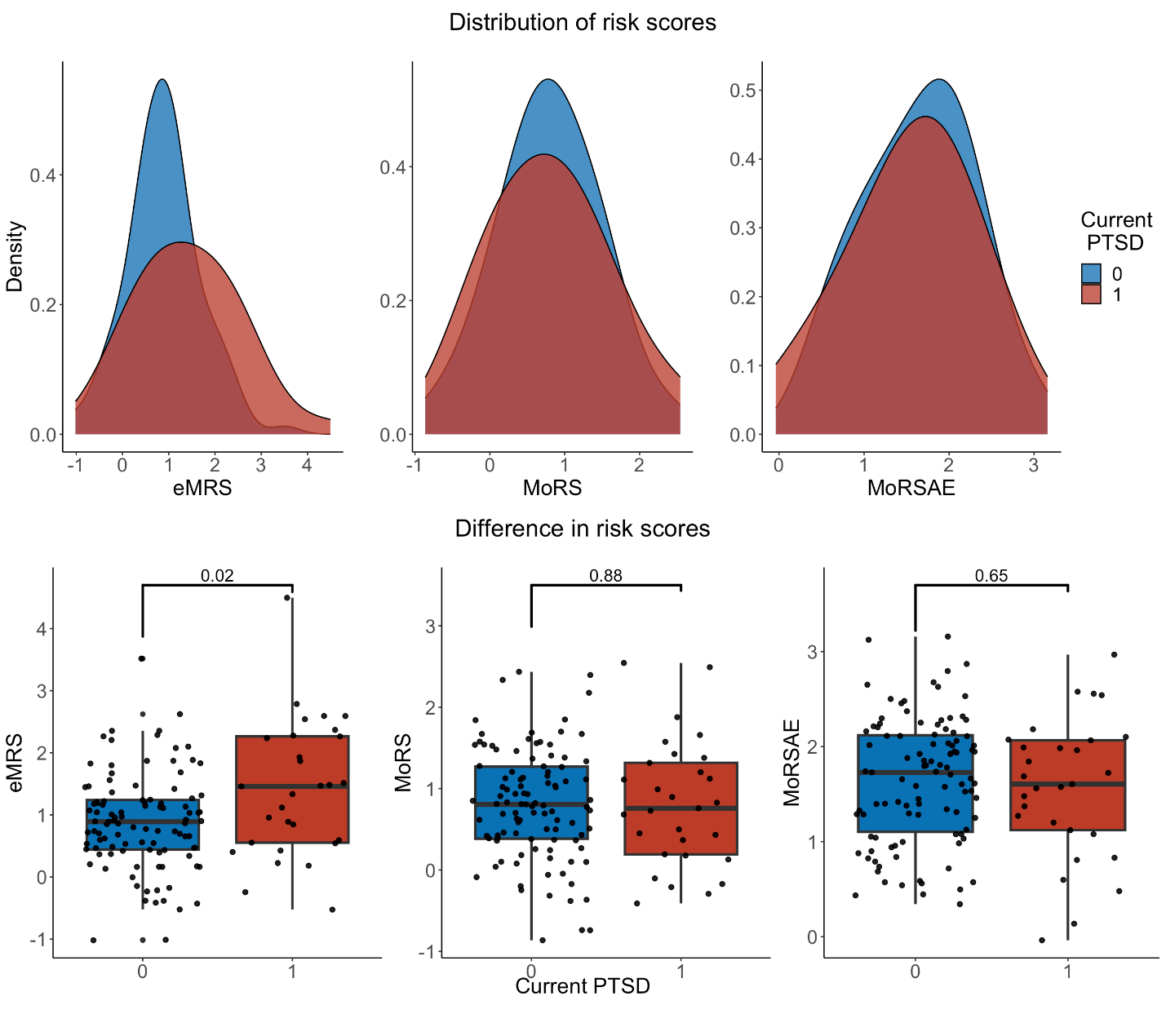


**Figure S5**: BEAR Cohort (N = 132)—distribution and difference in risk scores. A significant difference was found in eMRS between cases and controls. No significant difference was found in MoRS and MoRSAE between cases and controls.


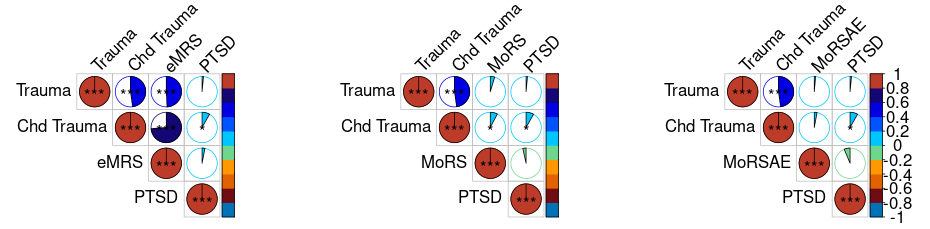


**Figure S6**: NCPTSD-TRACTS (N = 941)—Correlation between continuous variables was analyzed using Pearson's correlation. Point-Biserial correlation was used correlation between continuous variables and PTSD. No significant correlation was observed between eMRS, MoRS, MoRSAE and PTSD.


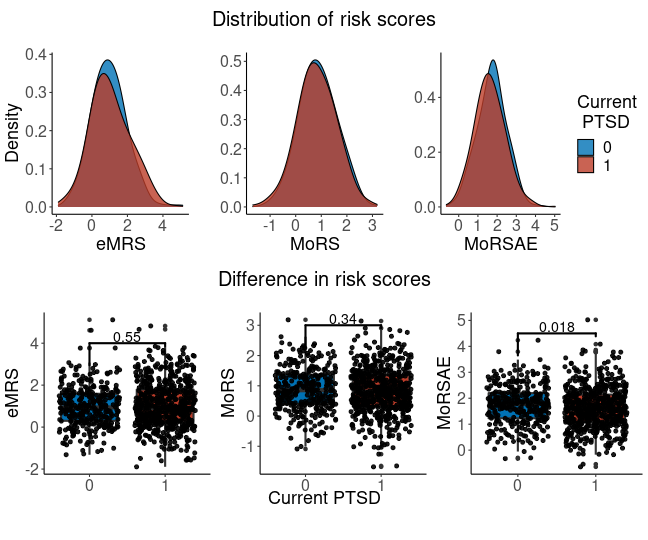


**Figure S7**: NCPTSD-TRACTS (N = 941)—distribution and difference in risk scores. No significant difference was found in eMRS and MoRS between cases and controls. A significant difference was found in MoRSAE between cases and controls.


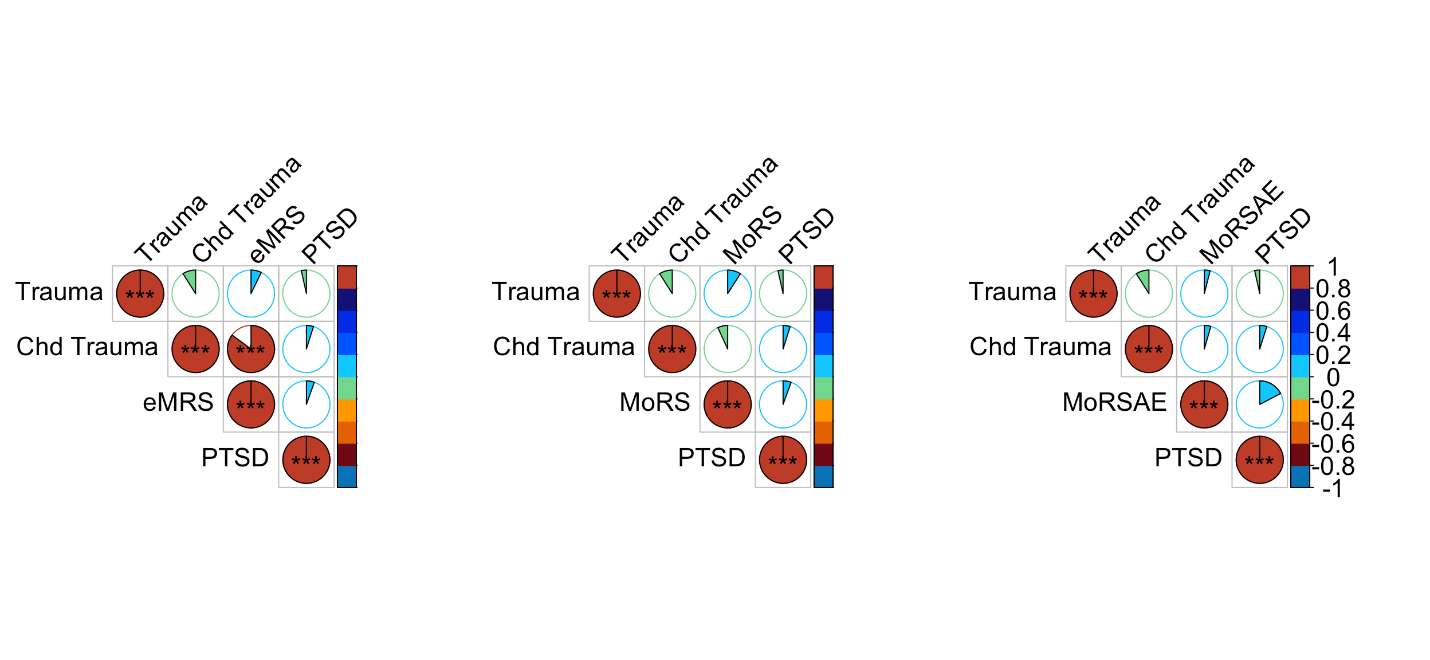


**Figure S8**: PROGrESS cohort (N = 130)—Correlation between continuous variables was analyzed using Pearson's correlation. Point-Biserial correlation was used correlation between continuous variables and PTSD. No significant correlation was observed between eMRS, MoRS, MoRSAE and PTSD.


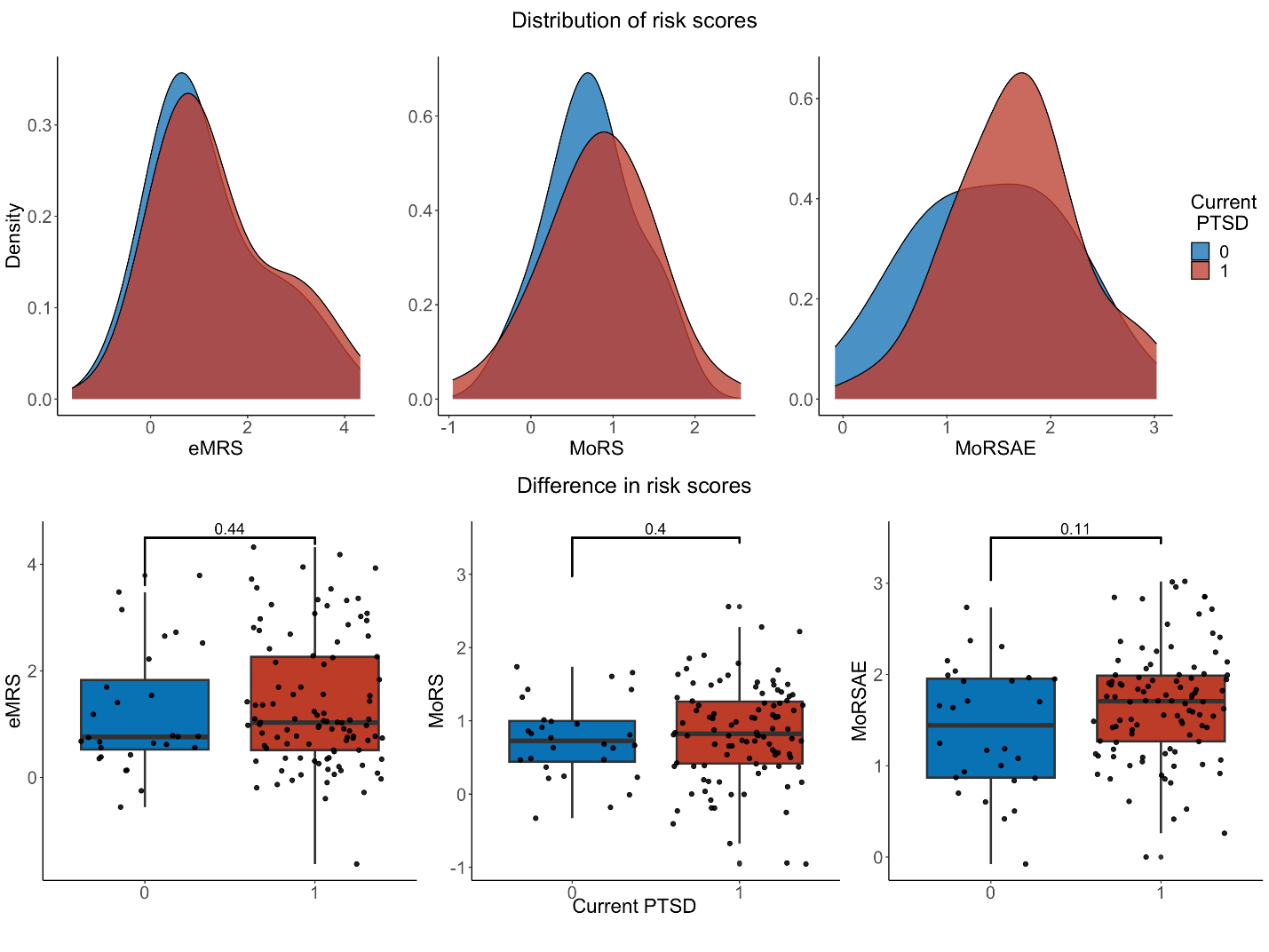
 **Figure S9**: PROGrESS cohort (N = 130)—distribution and difference in risk scores. No significant difference was found between cases and controls.


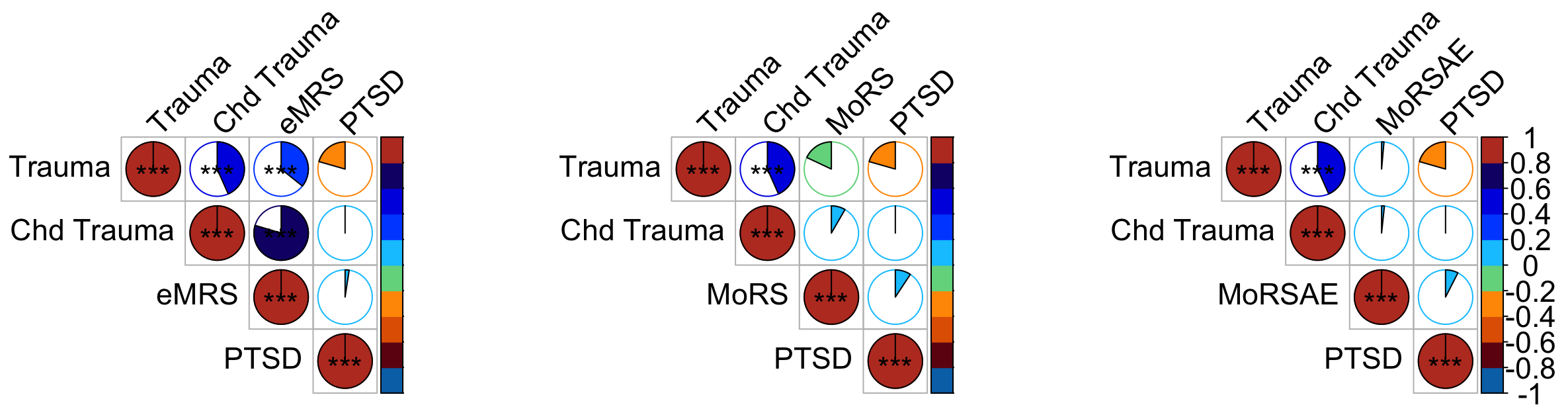


**Figure S10**: DCHS cohort (N = 91)—Correlation between continuous variables was analyzed using Pearson's correlation. Point-Biserial correlation was used correlation between continuous variables and PTSD. No significant correlation was observed between eMRS, MoRS, MoRSAE and PTSD.


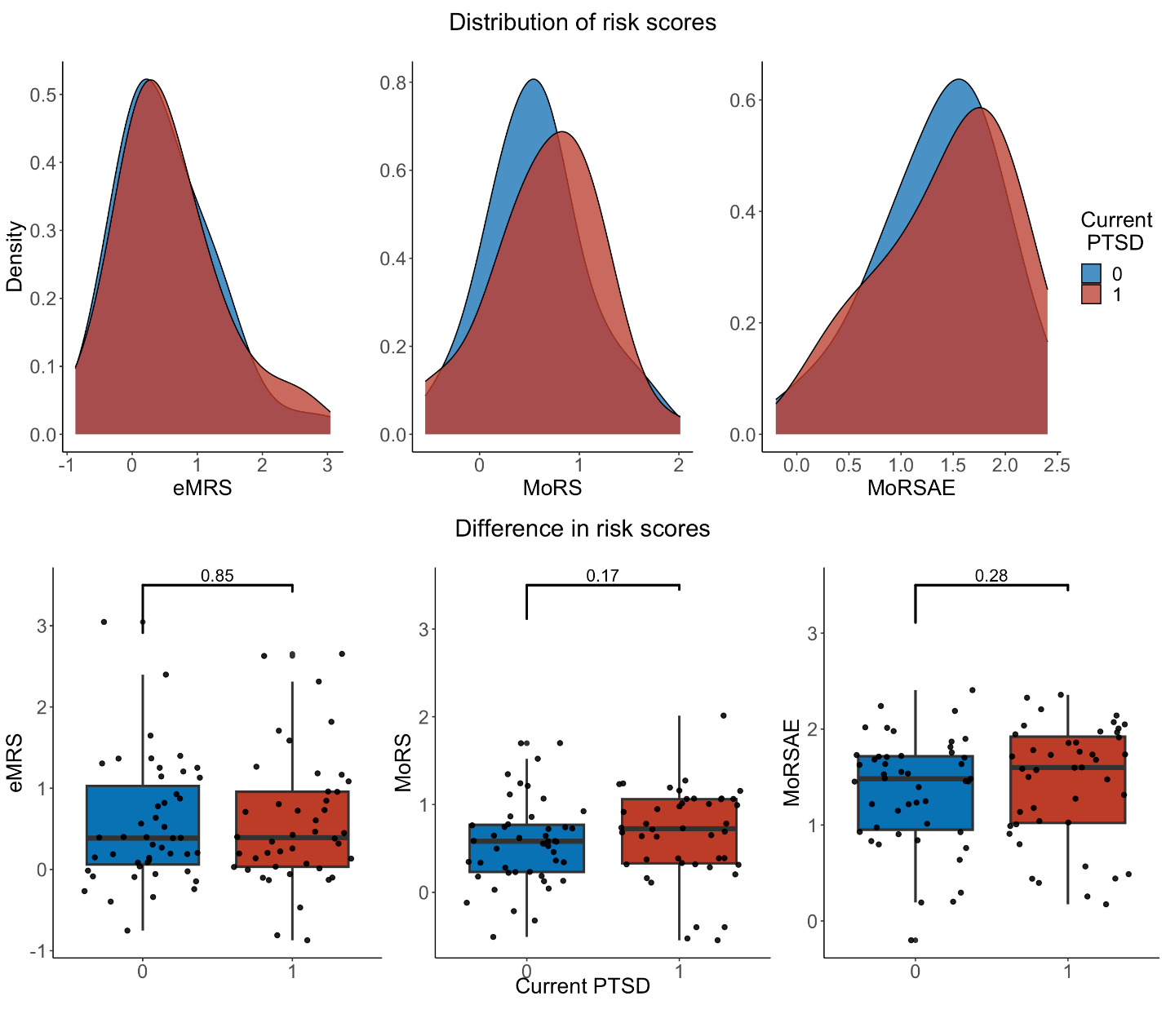


**Figure S11**: DCHS cohort (N = 91)— distribution and difference in risk scores. No significant difference was found in eMRS, MoRS and MoRSAE between cases and controls.

|  |  | **Current PTSD** | **Controls** |  |  |
| --- | --- | --- | --- | --- | --- |
| **Cohort (control, case)** | **Variable** | **Mean (SD)** | | **P value**  **(t test)** | **P value**  **(Mann-Whitney )** |
| Army STARRS (42, 111) | Cumulative Trauma | 1 (0) | 1 (0) | NA | NA |
|  | Childhood Trauma | 7.1(3.3) | 6.3(2.2) | 0.1438 | 0.2421 |
| DNHS (31, 385) | Cumulative Trauma | 12.2 (7) | 6.1 (4.1) | **3.63E-05** | **3.36E-08** |
|  | Childhood Trauma | 7.6(5.7) | 4.4(3.4) | **0.0044** | **0.0013** |
| GTP (161, 323) | Cumulative Trauma | 7 (3.1) | 4.4 (2.8) | **2.32E-17** | **1.95E-18** |
|  | Childhood Trauma | 56.1 (20.1) | 37.7 (13.4) | **1.66E-21** | **3.28E-25** |
| MRS (63, 60) | Cumulative Trauma | 11.2 (2.9) | 10.3 (3.8) | 0.1248 | 0.0674 |
|  | Childhood Trauma | 41.7 (12.2) | 37.5 (10.4) | **0.0410** | **0.0460** |
| PRISMO (17, 33) | Cumulative Trauma | 6.5 (3.1) | 5.9 (3.8) | 0.5576 | 0.5921 |
|  | Childhood Trauma | 5.5 (2.6) | 2.8 (2.2) | **0.0010** | **0.0007** |
| NCPTSD-TRACTS (609, 332) | Cumulative Trauma | 5.77 (4.41) | 5.64 (3.82) | 0.6248 | 0.8825 |
|  | Childhood Trauma | 1.29 (1.45) | 1.06 (1.20) | **0.0080** | 0.0965 |
| BEAR (103, 29) | Cumulative Trauma | 6.10 (3.17) | 4.17 (2.40) | **4.09E-03** | **3.87E-03** |
|  | Childhood Trauma | 59.86 (22.74) | 44.90 (13.23) | **1.83E-03** | **5.37E-04** |
| DCHS (47, 44) | Cumulative Trauma | 2.05 (1.64) | 2.96 (2.60) | **0.0477** | **0.0429** |
|  | Childhood Trauma | 36.36 (13.29) | 36.30 (12.74) | 0.9809 | 0.723 |
| PROGrESS (28, 102) | Combat Trauma | 20.35 (6.55) | 20.86 (2.99) | 0.5603 | 0.7832 |
|  | Childhood Trauma |  |  | 0.6459* |  |

Table S1: Cumulative and childhood trauma information

* Fisher test p-value for categorical variables

Table S2: Logistic regression summary in independent cohorts

| **Cohort** | **Model** | **Beta** | **Std. Error** | **Z value** | **pvalue** |
| --- | --- | --- | --- | --- | --- |
| NCPTSD-TRACTS | eMRS | 0.0598 | 0.0640 | 0.935 | 0.35 |
|  | MoRS | -0.0977 | 0.0915 | -1.068 | 0.286 |
|  | MoRSAE | -0.1707 | 0.0880 | -1.940 | 0.052 |
| BEAR | **eMRS** | **0.6839** | **0.2514** | **2.720** | **0.006** |
|  | MoRS | 0.0239 | 0.3020 | 0.079 | 0.937 |
|  | MoRSAE | -0.1824 | 0.3135 | -0.582 | 0.5607 |
| DCHS | eMRS | 0.06318 | 0.27675 | 0.228 | 0.819 |
|  | MoRS | 0.3739 | 0.4231 | 0.884 | 0.377 |
|  | MoRSAE | 0.2619 | 0.3651 | 0.717 | 0.473 |
| PROGrESS | eMRS | 0.1141 | 0.1834 | 0.622 | 0.533 |
|  | MoRS | 0.2156 | 0.3390 | 0.636 | 0.524 |
|  | MoRSAE | 0.6827 | 0.3512 | 1.944 | 0.051 |

References

1. Goldmann E, Aiello A, Uddin M, et al. Pervasive exposure to violence and posttraumatic stress disorder in a predominantly African American Urban Community: the Detroit Neighborhood Health Study*.* *Journal of traumatic stress* 24, 747-51 (2011).

2. Uddin M, Aiello AE, Wildman DE, et al. Epigenetic and immune function profiles associated with posttraumatic stress disorder*.* *Proceedings of the National Academy of Sciences of the United States of America* 107, 9470-5 (2010).

3. Breslau N, Kessler RC, Chilcoat HD, Schultz LR, Davis GC, and Andreski P. Trauma and posttraumatic stress disorder in the community: the 1996 Detroit Area Survey of Trauma*.* *Archives of general psychiatry* 55, 626-32 (1998).

4. *Diagnostic and statistical manual of mental disorders, 4th ed*. Diagnostic and statistical manual of mental disorders, 4th ed. 1994, Arlington, VA, US: American Psychiatric Publishing, Inc. xxvii, 886-xxvii, 886.

5. Straus MA. Measuring Intrafamily Conflict and Violence: The Conflict Tactics (CT) Scales*.* *Journal of Marriage and Family* 41, 75-88 (1979).

6. Bernstein DP, Ahluvalia T, Pogge D, and Handelsman L. Validity of the Childhood Trauma Questionnaire in an Adolescent Psychiatric Population*.* *Journal of the American Academy of Child & Adolescent Psychiatry* 36, 340-348 (1997).

7. Keyes KM, McLaughlin KA, Koenen KC, Goldmann E, Uddin M, and Galea S. Child maltreatment increases sensitivity to adverse social contexts: Neighborhood physical disorder and incident binge drinking in Detroit*.* *Drug and Alcohol Dependence* 122, 77-85 (2012).

8. Gillespie CF, Bradley B, Mercer K, et al. Trauma exposure and stress-related disorders in inner city primary care patients*.* *General Hospital Psychiatry* 31, 505-514 (2009).

9. Binder EB, Bradley RG, Liu W, et al. Association of FKBP5 polymorphisms and childhood abuse with risk of posttraumatic stress disorder symptoms in adults*.* *Jama* 299, 1291-1305 (2008).

10. Bernstein DP, Fink L, Handelsman L, Foote JJAofvAhfr, and practitioners. Childhood trauma questionnaire*.* (1998).

11. Blake DD, Weathers FW, Nagy LM, et al. The development of a clinician-administered PTSD scale*.* 8, 75-90 (1995).

12. Weathers FW, Keane TM, Davidson JRJD, and anxiety. Clinician‐Administered PTSD Scale: A review of the first ten years of research*.* 13, 132-156 (2001).

13. Ursano RJ, Colpe LJ, Heeringa SG, Kessler RC, Schoenbaum M, and Stein MB. The Army study to assess risk and resilience in servicemembers (Army STARRS)*.* *Psychiatry* 77, 107-19 (2014).

14. Stein MB, Campbell-Sills L, Ursano RJ, et al. Childhood Maltreatment and Lifetime Suicidal Behaviors Among New Soldiers in the US Army: Results From the Army Study to Assess Risk and Resilience in Servicemembers (Army STARRS)*.* *The Journal of clinical psychiatry* 79, 16m10900 (2018).

15. Kessler RC, Santiago PN, Colpe LJ, et al. Clinical reappraisal of the Composite International Diagnostic Interview Screening Scales (CIDI-SC) in the Army Study to Assess Risk and Resilience in Servicemembers (Army STARRS)*.* *International journal of methods in psychiatric research* 22, 303-21 (2013).

16. Nievergelt CM, Maihofer AX, Mustapic M, et al. Genomic predictors of combat stress vulnerability and resilience in U.S. Marines: A genome-wide association study across multiple ancestries implicates PRTFDC1 as a potential PTSD gene*.* *Psychoneuroendocrinology* 51, 459-71 (2015).

17. Baker DG, Nash WP, Litz BT, et al. Predictors of risk and resilience for posttraumatic stress disorder among ground combat Marines: methods of the Marine Resiliency Study*.* *Preventing chronic disease* 9, E97 (2012).

18. Reijnen A, Rademaker AR, Vermetten E, and Geuze E. Prevalence of mental health symptoms in Dutch military personnel returning from deployment to Afghanistan: a 2-year longitudinal analysis*.* *European psychiatry : the journal of the Association of European Psychiatrists* 30, 341-6 (2015).

19. Eekhout I, Reijnen A, Vermetten E, and Geuze E. Post-traumatic stress symptoms 5 years after military deployment to Afghanistan: an observational cohort study*.* *The lancet. Psychiatry* 3, 58-64 (2016).

20. Mirjam van Zuiden, M.Sc. ,, Elbert Geuze, Ph.D. ,, Hanneke L.D.M. Willemen, M.Sc. ,, et al. Pre-Existing High Glucocorticoid Receptor Number Predicting Development of Posttraumatic Stress Symptoms After Military Deployment*.* 168, 89-96 (2011).

21. Bremner JD, Bolus R, Mayer EAJTJon, and disease m. Psychometric properties of the early trauma inventory–self report*.* 195, 211 (2007).

22. Hovens JE, Bramsen I, and van der Ploeg HM. Self-rating inventory for posttraumatic stress disorder: review of the psychometric properties of a new brief Dutch screening instrument*.* *Perceptual and motor skills* 94, 996-1008 (2002).

23. Nugent NR, Armey M, Boker S, et al. Adolescents hospitalised for suicidality: biomarkers, social and affective predictors: a cohort study*.* 12, e056063 (2022).

24. Logue MW, Baldwin C, Guffanti G, et al. A genome-wide association study of post-traumatic stress disorder identifies the retinoid-related orphan receptor alpha (RORA) gene as a significant risk locus*.* *Mol Psychiatry* 18, 937-42 (2013).

25. McGlinchey RE, Milberg WP, Fonda JR, and Fortier CB. A methodology for assessing deployment trauma and its consequences in OEF/OIF/OND veterans: The TRACTS longitudinal prospective cohort study*.* *International journal of methods in psychiatric research* 26, (2017).

26. Blake DD, Weathers FW, Nagy LM, et al. The development of a Clinician-Administered PTSD Scale*.* *Journal of traumatic stress* 8, 75-90 (1995).

27. Weathers FW, Bovin MJ, Lee DJ, et al. The Clinician-Administered PTSD Scale for DSM-5 (CAPS-5): Development and initial psychometric evaluation in military veterans*.* *Psychol Assess* 30, 383-395 (2018).

28. Kubany ES, Haynes SN, Leisen MB, et al. Development and preliminary validation of a brief broad-spectrum measure of trauma exposure: the Traumatic Life Events Questionnaire*.* *Psychol Assess* 12, 210-24 (2000).

29. Rauch SAM, Kim HM, Powell C, et al. Efficacy of Prolonged Exposure Therapy, Sertraline Hydrochloride, and Their Combination Among Combat Veterans With Posttraumatic Stress Disorder: A Randomized Clinical Trial*.* *JAMA Psychiatry* 76, 117-126 (2019).

30. Rauch SAM, Simon NM, Kim HM, et al. Integrating biological treatment mechanisms into randomized clinical trials: Design of PROGrESS (PROlonGed ExpoSure and Sertraline Trial)*.* *Contemp Clin Trials* 64, 128-138 (2018).

31. Stein DJ, Koen N, Donald KA, et al. Investigating the psychosocial determinants of child health in Africa: The Drakenstein Child Health Study*.* *J Neurosci Methods* 252, 27-35 (2015).

32. Lecrubier Y, Sheehan DV, Weiller E, et al. The Mini International Neuropsychiatric Interview (MINI). A short diagnostic structured interview: reliability and validity according to the CIDI*.* *European psychiatry* 12, 224-231 (1997).

33. Sheehan DV, Lecrubier Y, Sheehan KH, et al. The Mini-International Neuropsychiatric Interview (M.I.N.I): The development and validation of a structured diagnostic psychiatric interview for DSM-IV and ICD-10*.* *The Journal of Clinical Psychiatry* 59, 22-33 (1998).
